# Early-life intraguild predation risk produces adaptive personalities in predatory mites

**DOI:** 10.1016/j.isci.2024.109065

**Published:** 2024-02-01

**Authors:** Peter Schausberger, Thi Hanh Nguyen, Mustafa Altintas

**Affiliations:** 1 Department of Behavioral and Cognitive Biology, University of Vienna, Djerassiplatz 1, Vienna 1030, Austria

**Keywords:** Biological sciences, Zoology, Evolutionary biology

## Abstract

Animal personalities are defined by within-individual consistency, and consistent among-individual variation, in behavior across time and/or contexts. Here we hypothesized that brief early-life experience of intraguild predation (IGP) risk has enduring phenotypic effects on personality expression in boldness and aggressiveness in later life. We tested our hypothesis in predatory mites *Phytoseiulus persimilis*, which are IG predators with ontogenetic role reversals, i.e., they are potential IG prey during early life but IG predators as adults. Adult *P. persimilis* females, which had experienced IGP risk early in life or not, were subjected to three tests each for boldness and aggressiveness. IGP-experienced individuals were on average bolder and more aggressive. Boldness was moderately repeatable, aggressiveness was weakly repeatable. Strikingly, early-life IGP experience shifted the within-group personality composition toward consistently bold and aggressive personalities. Phenotypic adjustment of personality expression was adaptive, as indicated by the positive correlation between personality scores and egg production.

## Highlights

• Animal personalities abound yet the pathways mediating personalities remain elusive
• Early-life experience of intraguild predation risk changes adult mite personalities
• Risk-experienced individuals are bolder and more aggressive than risk-naive individuals
• Bolder and more aggressive personalities produce more eggs

## Graphical abstract

**Figure:**
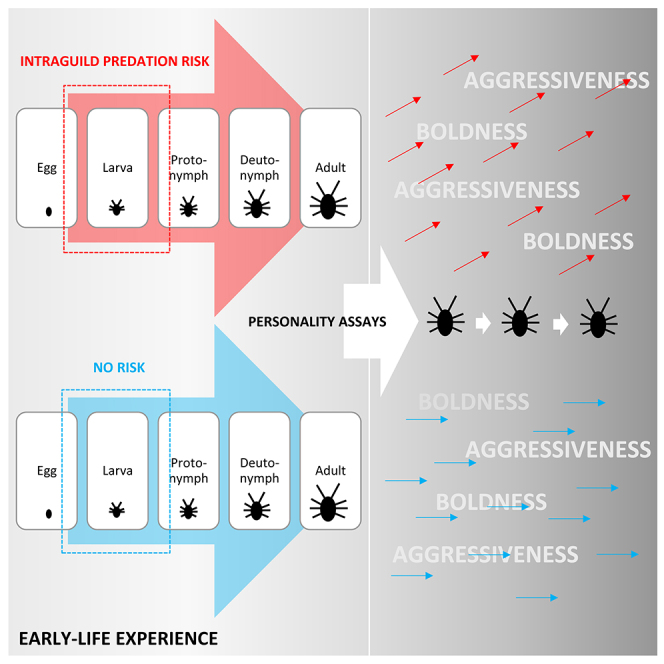

## Introduction

Early-life experiences are major determinants of behavioral trajectories, including animal personality formation and adult phenotypes.^1^^,^^2^^,^^3^ Traumatic experiences such as exposure to predation risk during early life have the potential to permanently shape adult phenotypes and personalities.^4^ Typical personality traits affected by early-life predation risk are boldness, that is, risk-taking behavior in familiar environments, and aggressiveness, that is, hostile behaviors directed toward conspecific individuals.^5^ Boldness and aggressiveness are often part of a behavioral syndrome (suite of correlated behavioral traits), i.e., they covary across individuals due to links in the underlying genetic architecture.^5^ While several studies showed that predation on young and/or immature individuals may select for bolder or shier older and/or mature individuals in a population,^6^^,^^7^^,^^8^ plastic phenotypic changes in personalities following predation risk experience early in life remain elusive. Also, most pertinent studies looked at mean behavioral tendencies in a population but did not look at within-individual consistencies.^6^^,^^7^^,^^8^ For example, zebrafish from a high-predation population were on average bolder than those from a low-predation population.^6^ Wild bighorn sheep ewes became, on average, bolder due to selective predation of shy ewes by cougars.^7^ The opposite, predators selecting against boldness in prey, was observed in trout.^8^ Similarly, perch from predator-free populations were bolder than those from predator-exposed populations^9^ though this result may have been confounded by other population differences. Bell and Sih (2007)^10^ quantified the relative influence of selection and plasticity, following predator exposure, on induction of a boldness/aggressiveness syndrome in adult sticklebacks; noteworthy, the sticklebacks did not change in boldness, just the syndrome became apparent. Urszan et al. (2015)^11^ observed personality formation in tadpoles following experience with predator cues, though no real predation risk, early in life. Without early-life predator cue exposure, the tadpoles’ behavioral traits were not repeatable, that is, the tadpoles did not show personalities.

**Figure 1:**
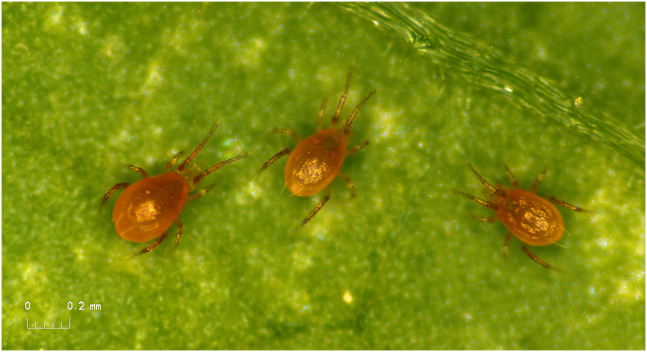
Adult predatory mite females, *Phytoseiulus persimilis*, on a bean leaf.

Here, we assessed the influence of ephemeral intraguild predation (IGP) risk early in life (in the postnatal phase) on the expression of personalities in boldness and aggressiveness as adults in plant-inhabiting predatory mites *Phytoseiulus persimilis* (Figure 1). These mites are ideal model animals to address our research questions. *P. persimilis* are specialized predators of herbivorous spider mites (Tetranychidae) and, like most predatory mites of the family Phytoseiidae, engage in cannibalism and IGP.^12^^,^^13^^,^^14^ Their development proceeds from egg to larva over two nymphal stages (proto- and deutonymph) to adult. Predatory mites lack eyes and sense their environment primarily via chemical (olfactory and tactile) and mechanical sensory modalities.^15^ Early-life experiences by *P. persimilis* can induce persistent behavioral changes in predator-prey and social interactions.^16^^,^^17^^,^^18^ IGP among predatory mites is typically mutual but asymmetric, in dependence of the life stages involved and diet specialization.^12^^,^^13^ Larvae are prone to become IG prey of co-occurring predatory mites, usually those with generalist feeding habits^19^; adult mites, especially gravid females, are becoming IG predators themselves and are no longer prone to be attacked by others.^12^^,^^13^ *P. persimilis* are threat-sensitive, i.e., they can distinguish between low and high-risk IG predators and adjust their behaviors accordingly.^12^^,^^18^^,^^20^

While there exist a few studies on the effects of the risk of classical predation on personality expression,^10^^,^^11^ the effects of IGP risk experience early in life on adult personalities are unknown. Considering the widespread occurrence and eco-evolutionary relevance of IGP,^19^ scrutinizing links between IGP and the formation and expression of animal personalities are highly important tasks. From a functional perspective, IGP is a more complex interaction than classical predation.^19^ IGP differs from classical predation because of multi-functionality (eliminating a resource competitor, obtaining nutrients, maternal care by eliminating a predator of offspring), the combined selective forces of predation and competition acting on IG members, and commonly occurring ontogenetic role reversals (this is also the case in predatory mites^12^^,^^13^^,^^18^). Any shifts in adult behavior due to IGP risk early in life are linked to ontogenetic role reversals because the environmental stimuli (here of IGP risk) are perceived as IG prey but the changes manifest in later stages that are IG predators. Only the offspring of the IG predators become again potential IG prey. Thus, in mutual IGP systems, such as in a guild of co-occurring predatory mite species sharing a plant, adult IG predators should not refrain from exploiting resources located in risky sites and killing IG prey; however, it pays to minimize the IGP risk to offspring by oviposition in safe sites.^18^^,^^20^ In addition to the effects of early-life experience of IGP risk on personality formation, we were interested in potential links between personality expression and fitness. Though trends have been identified that bolder and more aggressive individuals have higher reproductive success,^21^ the adaptive value of environmentally induced shifts in personality expression, following experience of classical predation or IGP risk early in life, has not yet been examined.

## Results

The number of individuals surviving to adulthood in the conditioning leaf arenas was about 20% lower in groups of *P. persimilis* exposed to the IG predator *A. andersoni* than in unexposed groups (mean ± SE; IGP-naive total 12.57 ± 0.57, females 9.35 ± 0.59; IGP-experienced total 9.92 ± 0.53, females 7.07 ± 0.53; generalized linear models [GLM] total *Wald Ӽ*^*2*^ = 16.356, p = 0.001; GLM females *Wald Ӽ*^*2*^ = 9.366, p = 0.002). Lacking correlation (Pearson product-moment) between the number of surviving individuals in the conditioning leaf arenas and the personality scores in boldness and aggressiveness indicates random IGP (boldness: *N* = 70, *r* = −0.052, p = 0.662; aggressiveness: *N* = 113, *r* = −0.035, p = 0.717). Less than 100% survival in unexposed groups was due to natural mortality typically occurring in the used type of leaf arena.

**Figure 2:**
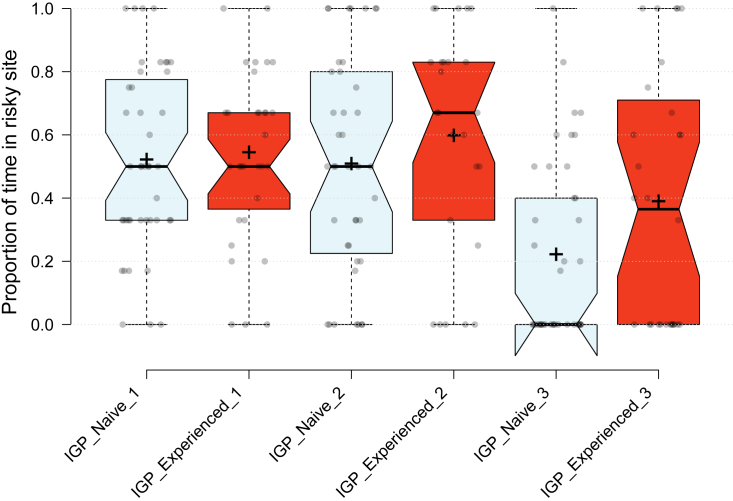
Residence preference of IGP-naive and IGP-experienced females of *P persimilis* in three successive boldness tests, each representing a different context In each test, each female was given a choice between safe and risky sites in an acrylic T-maze cage. Thick horizontal lines are the medians, pluses show the means, boxes represent the interquartile range, whiskers extend to minimum and maximum values, light gray dots are the individual data points. GLM: p = 0.04 between IGP-naive and IGP-experienced females across tests.

**Figure 3:**
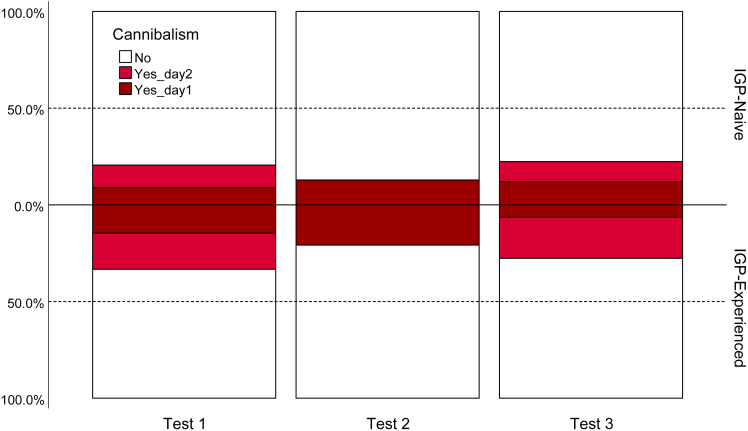
Occurrence of cannibalism of larvae by *P. persimilis* females in three successive aggressiveness tests, each representing a different context In each of the three tests, each female was offered one conspecific larva as potential prey inside an acrylic cage and monitored for cannibalism of the larva over 7 h on day 1 and, in tests 1 and 3, again after 24 h on day 2). GLM: p = 0.03 between IGP-naive (N = 70) and IGP-experienced (N = 48) females across tests.

**Figure 4:**
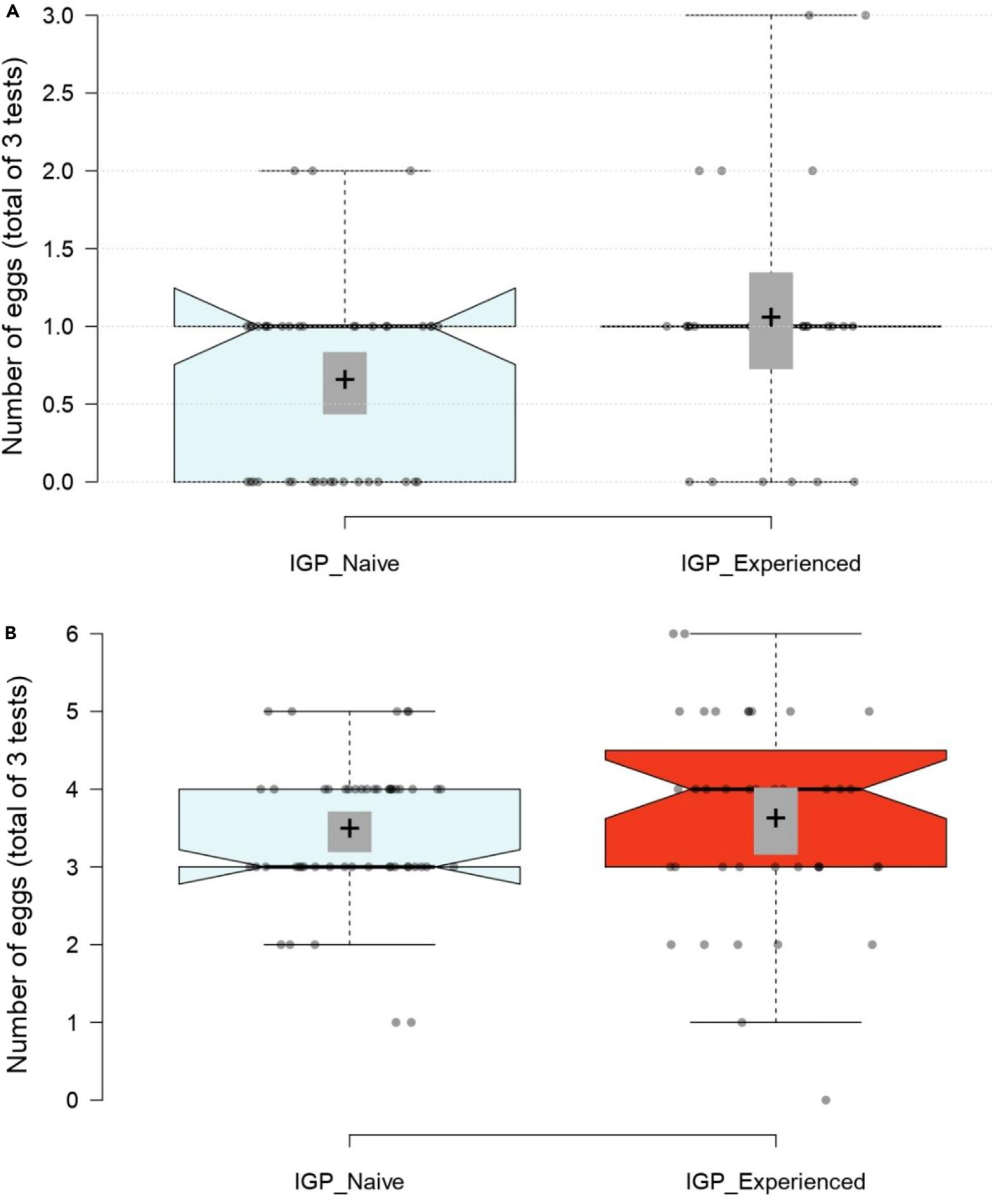
Egg production by IGP-naive and IGP-experienced *P. persimilis* females Total number of eggs laid by IGP-naive and IGP-experienced females during testing for boldness (A) and aggressiveness (B) (three tests for each trait). Thick horizontal lines are the medians, pluses and associated gray areas show the means ±1 SE, boxes represent the interquartile range, whiskers extend to minimum and maximum values, light gray dots are the individual data points. GLM: p = 0.03 in the boldness assay (A) and p = 0.56 in the aggressiveness assay (B).

**Figure 5:**
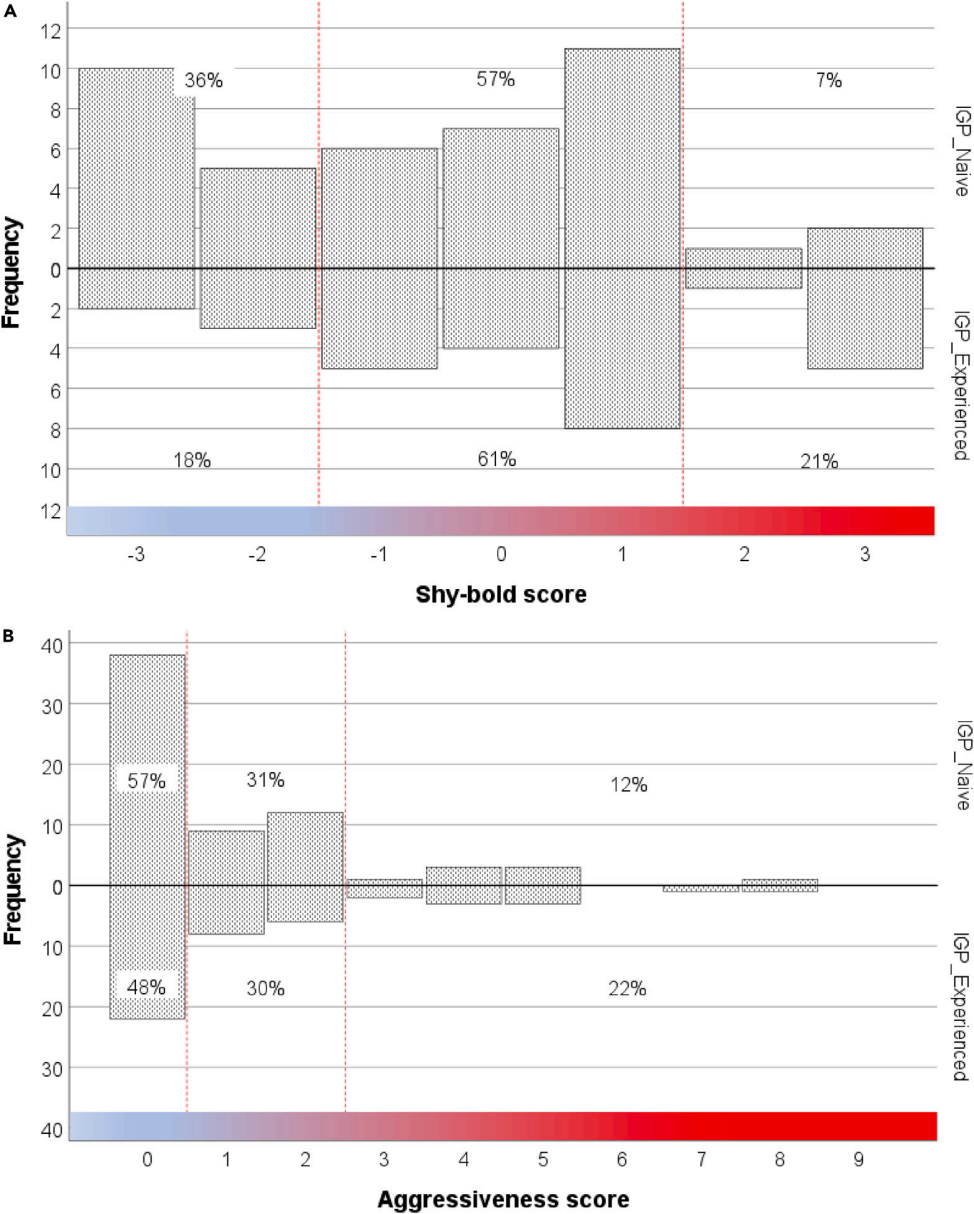
Composition of personality types within groups of IGP-naive and IGP-experienced *P. persimilis* females. Frequency distribution of personality types in boldness (A) and aggressiveness (B). Percentages refer to the relative frequency of personality types within sectors, with higher scores indicating bolder or more aggressive individuals (see Table S1 for categorization of types) and the marginal sectors comprising the behaviorally consistent individuals. The personality composition differed between early-life treatments (IGP-naive and IGP-experienced) in both traits (GLM; p ≤ 0.05). See also Table S1.

**Figure 6:**
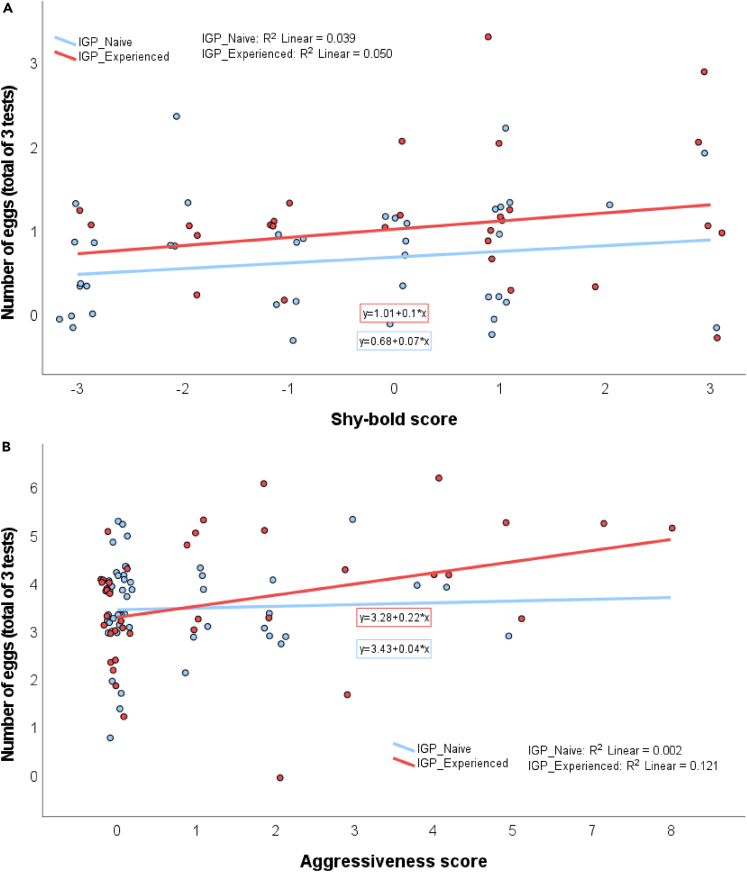
Relationship between egg production and personality types of IGP-naive and IGP-experienced *P. persimilis* females Linear regression of eggs laid on personality types of *P. persimilis* in boldness (A) and aggressiveness (B). Pooled *R*^*2*^ = 0.068, p = 0.031 for boldness (N = 41, p = 0.216 for IGP-naive and N = 28, p = 0.253 for IGP-experienced) and *R*^*2*^ = 0.054, p = 0.016 for aggressiveness (N = 51, p = 0.734 for IGP-naive and N = 39, p = 0.028 for IGP-experienced). See also Table S1.

IGP-experienced predators were on average bolder (GEE: *Wald Ӽ*^*2*^ = 4.006, p = 0.045; Figure 2) and more aggressive (*Wald Ӽ*^*2*^ = 4.270, p = 0.039; Figure 3) than IGP-naive predators. The mean number of eggs produced over three tests was higher in IGP-experienced than IGP-naive predators (Figure 4), which was statistically significant in the boldness experiment (GLM: *Wald Ӽ*^*2*^ = 5.641, p = 0.018) but not in the aggressiveness experiment (*Wald Ӽ*^*2*^ = 0.306, p = 0.580). In the boldness experiment, predators of both early-life treatments placed most of their eggs away from the risky site (Wilcoxon: IGP-naive *Z* = −2.400, p = 0.016; IGP-experienced *Z* = −2.668, p = 0.008). Intraclass correlation coefficients (ICC) revealed moderate repeatability in boldness, and weak, though significant, repeatability in aggressiveness (Table 1). Treatment-specific ICCs in boldness were higher in IGP-naive than IGP-experienced predators, which was mainly due to IGP experience decreasing within-individual consistency. The opposite was true for treatment-specific ICCs in aggressiveness (higher in IGP-experienced than IGP-naive predators), which was caused by a concurrent increase of within-individual consistency and among-individual variability. However, in neither trait did the treatment-specific ICCs statistically differ from each other and the overall ICC, as evident from the widely overlapping 95% confidence intervals (Table 1). Personality composition was significantly influenced by early-life experience of IGP risk (GLM boldness: *Wald Ӽ*^*2*^ = 4.652, p = 0.031; GLM aggressiveness: *Wald Ӽ*^*2*^ = 3.849, p = 0.050), with IGP risk experience shifting the personalities toward bolder and more aggressive individuals (Figure 5). Linear regression revealed that bolder (*R*^*2*^ = 0.068, p = 0.031) and more aggressive (*R*^*2*^ = 0.054, p = 0.016; primarily in the IGP-experienced treatment) personalities also laid more eggs (Figure 6).

**Table 1:** Intraclass correlation coefficients (ICC; two-way random, consistency) in boldness (proportion of time spent in risky site in three contexts; average measure) and aggressiveness (propensity for cannibalism of non-kin in three contexts; single measure) of *P persimilis* females in dependence of early-life experience (yes/no) of risk

Personality trait	Early-life treatment	N	ICC	CI (95%)	p value
Boldness (time in risky site)	IGP-naive	39	0.426^b^	0.023; 0.679	0.020^b^
	IGP-experienced	25	0.257	−0.444; 0.649	0.187
	Pooled	64	0.361^b^	0.033; 0.591	0.017^b^
Aggressiveness^a^ (attack yes/no)	IGP-naive	66	0.094	−0.045; 0.257	0.096
	IGP-experienced	46	0.183^b^	0.008; 0.382	0.020^b^
	Pooled	112	0.140^b^	0.028; 0.264	0.006^b^
Aggressiveness (attack latency)	IGP-naive	66	0.051	−0.082; 0.211	0.235
	IGP-experienced	46	0.163^b^	−0.010; 0.361	0.033^b^
	Pooled	112	0.086	−0.021; 0.208	0.060

a Alternative analysis using Fleiss’ Kappa (Κ) and “rptR” for binary data (R) resulted basically in the same outcome: K = 0.088 (R = 0.085) and p = 0.215 (p = 0.115) for IGP-naive, K = 0.173 (R = 0.159) and p = 0.042 (p = 0.042) for IGP-experienced, K = 0.133 (R = 0.121) and p = 0.014 (p = 0.015) for overall.

b Significant ICCs and associated p values; N is the number of replicates.

## Discussion

Our experiments document that brief early-life experience of IGP risk exerts persistent effects, over two to three molting events, on mean expression of, as well as personality formation in, aggressiveness and boldness of plant-inhabiting predatory mites *P. persimilis*. IGP-experienced predators were on average bolder and more aggressive, and laid more eggs, than IGP-naive predators. Boldness was moderately repeatable, aggressiveness was weakly repeatable. While the behavioral consistencies (repeatability values) remained similar following early-life treatments (IGP experience versus IGP naiveté), early-life IGP experience changed the distribution of personality types along the shy-bold and aggressiveness axes. Personality scores in boldness and aggressiveness were positively correlated with egg production, which points at the adaptive value of phenotypic adjustment of personality expression to early-life experience.

Analogous to our study, Brown et al. (2007)^22^ observed that poeciliid fish became, on average, bolder after experiencing a threat, chasing with a net, during ontogeny (these authors did not assess personalities). Seiter and Schausberger (2015)^23^ showed that offspring of IGP-exposed mothers were, as juveniles, on average bolder than offspring of IGP-naive mothers (these authors did not test for personalities). A few studies, such as Bell and Sih (2007)^10^ for fish and Urszan et al. (2015)^11^ for tadpoles, looked at experience-induced (though not early-life on adult) changes in boldness repeatability, i.e., whether predation risk induced or eradicated the formation of personalities or behavioral syndromes. Our findings highlight the critical relevance of ephemeral postnatal experiences for both mean and individual expression of behavioral traits and for changes in within-group personality composition. The latter is seldom looked at in animal personality research but important because repeatability is just an indicator of the occurrence of personalities within a group but does not tell about the within-group composition of personality types. Also, changes in the arithmetic mean of a given personality trait do not inform about changed composition because changed means may be the result of a general increase or decrease in the expression of a given trait or of a changed composition; very differently composed groups regarding personality types may have similar mean trait expressions or repeatability values.

Shifts in personality composition within groups may theoretically be due to selection (a given behavioral type is preferentially killed by the IG predators) or represent induced phenotypic plasticity (including changed reaction norms) or a combination of both. These alternative proximate explanations are an inherent property of every study design that exposes the experimental animals to real lethal risk, i.e., physical presence of predators, which represents the strongest possible predation risk stimulus. Study designs that expose the experimental animals only to predator cues, such as their smells or visual appearance, or to artificial models, can be ambiguous and difficult to interpret because prey may learn (habituation) that the cues alone are not accompanied by any real predation threat. Moreover, memory retention of predator cues alone may differ between shy and bold personalities, as shown for trout.^24^ For the following lines of evidence, we consider induced phenotypic plasticity as the prime, if not exclusive, cause of the observed shifts in personality composition in our experiments: (1) lacking correlation between the number of survivors in the conditioning arenas and personality scores indicates random IGP; (2) following early-life IGP risk experience, the frequency of consistently very bold and very aggressive individuals increased; some individuals became consistently bolder or more aggressive and others gave up consistent shyness or docility following IGP risk experience early in life; and (3) the treatment-specific ICC values for boldness and aggressiveness, respectively, were similar; if the observed shifts in the relative composition of personality types would have been due to selection, the ICC in the IGP-experienced treatment would have gone down; predators removing a given consistent type from the population, without concurrent plastic shifts toward consistency in others, would have compromised repeatability. However, in our experiments, the repeatability estimates stayed at about the same level in groups that experienced IGP risk early in life and those not.

Our study design, use of separate individuals and experiments for boldness and aggressiveness, does not allow to assess the boldness/aggressiveness syndrome at the individual level but the concurrent increase in mean boldness and mean aggressiveness levels and parallel shifts in personality composition in IGP-experienced individuals point at such a syndrome.^10^ Boldness-aggressiveness syndromes can be purely environmentally induced, as observed in sticklebacks,^10^ or can have a genetic basis, such as determined by the alleles on a single locus in zebrafish.^25^ In our experiments, IGP experience induced parallel shifts along the boldness and aggressiveness personality axes but this was not the cause of personality formation *per se* (repeatabilities were similar in IGP-naive and IGP-experienced predators), which indicates gene by environment interactions.

Bolder and more aggressive individuals having higher reproductive success seems a widespread pattern, though this advantage may come at the cost of shorter survival times, according to the meta-analysis by Smith and Blumstein (2008^21^; but see Moiron et al. 2020^26^ for a different conclusion regarding survival). Similar predictions are made by the hypothesis of the pace-of-life syndrome and its links to fitness.^7^^,^^27^ Higher fitness (mating success) of bolder individuals was, for example, observed in a free-ranging population of monitor lizards.^28^ Proximately, bolder individuals may benefit from enhanced access to resources but they may also compensate for their risk-prone behaviors by, for example, more pronounced (more successful) inducible defenses in morphology or anti-predator behavior.^29^ Regarding personalities in aggressiveness, the positive correlation with egg production was only true for IGP-experienced individuals. Higher reproduction by more aggressive individuals was not a function of obtaining a conspecific meal or not (every individual was just offered one conspecific prey item and an exclusive cannibalistic diet does not allow egg production in *P. persimilis*^13^^,^^14^) but rather indicates higher stress resilience and more favorable energy management in intraspecific competition.^30^ In either situation, IGP and cannibalism, higher and/or earlier oviposition should promote short-term fitness.^31^ Increased boldness and aggressiveness by adult females following IGP risk experience early in life observed in our experiments seem particularly advantageous in IGP scenarios because, due to ontogenetic role reversals, the females may act as IG predators of immature predatory mites but are themselves not at risk to be attacked.^12^^,^^13^ It thus pays, except for egg deposition, to not shy away from sites that signal IG predator presence but to go for them and try to eliminate resource competitors and potential future predators of offspring.

### Limitations of the study

Our study documents plastic shifts in personality expression in boldness and aggressiveness of adult individuals following short-term experience of IGP risk in early life. Future studies should address whether such shifts are exclusively induced when experience is made during a short, sensitive postnatal window, when during juvenile development the behavioral shifts start to emerge, and how long the personality shifts induced by early-life experience are apparent and maintained. Long-term and varied measures of fitness under circumstances other than the risk of IGP and conspecific aggression should allow a more inclusive picture of the fitness implications of early-life experience induced changes in personality formation and expression.

## STAR★Methods

### Key resources table

**Table:** 

REAGENT or RESOURCE	SOURCE	IDENTIFIER
**Experimental models: Organisms/strains**		

*Phytoseiulus persimilis*	Collected on open field aubergine in Sicily (this paper)	N/A
*Tetranychus urticae* (green form)	Collected on bean plants in a greenhouse in Vienna, Austria (this paper)	N/A
*Phaseolus vulgaris* var. Maxi BIO	Austrosaat	https://www.austrosaat.at/

**Software and algorithms**		

IBM SPSS version 28.0	IBM, Armonk, NY, USA	N/A
BoxPlotR	Spitzer et al. (2014)	http://shiny.chemgrid.org/boxplotr/

**Other**		

Leica M60	Leica Microsystems	https://www.leica-microsystems.com/
Leica M80	Leica Microsystems	https://www.leica-microsystems.com/

### Resource availability

#### Lead contact

Further information and requests for resources and reagents should be directed to and will be fulfilled by the lead contact, Peter Schausberger (peter.schausberger@univie.ac.at).

#### Materials availability

This study did not generate new unique reagents.

#### Data and code availability

• All data reported in this paper will be shared by the lead contact upon request.
• This paper does not report original code.
• Any additional information required to reanalyze the data reported in this paper is available from the lead contact upon request.

### Experimental model

#### Origin and rearing of experimental animals

*Phytoseiulus persimilis* females used in experiments derived from a laboratory-reared population that had been founded by specimens (∼300) collected from field-grown eggplant, *Solanum melongena*, in Palermo, Sicily. In the laboratory, *P. persimilis* was reared in heaps of common bean leaves infested by two-spotted spider mites, *Tetranychus urticae,* piled up on acrylic platforms. Each rearing unit consisted of a 14 × 14 × 0.2 cm platform resting on a water-soaked foam block (14 × 14 × 5 cm) inside a plastic box (20 × 20 × 6 cm) half-filled with tap water. Spider mite-infested leaves were added twice per week onto the platforms. To avoid contamination by other organisms, each rearing unit was placed in a plastic tray (40 × 30 × 8 cm), which bottom contained a thin layer of soapy water, and covered by a translucent acrylic case with a mesh-covered ventilation opening (10 cm Ø) on top. The predatory mites *Ambylseius andersoni* and *Neoseiulus californicus*, which were used as IG predators to condition *P. persimilis* early in life, or to produce risky sites in the boldness assay, were reared in similar rearing units as described above, with the only difference that they were fed a mixture of spider mites brushed from leaves into the arenas and *Typha* sp. pollen. All three predatory mite species used in this study share a co-evolutionary history. They co-occur, among other regions, in the Mediterranean basin^32^ and are mutual IG predators.^18^^,^^20^ The laboratory population of two-spotted spider mites, *T. urticae* (green form), had been founded by specimens (∼5000) collected on common bean plants *Phaseolus vulgaris* in a greenhouse in Vienna. In the laboratory, the spider mites were reared on whole common bean plants, *P. vulgaris* var. Maxi, grown in a peat-moss/sand mixture in 12 cm Ø pots. Both the predator rearing units and the bean plants were kept in an air-conditioned room at 23 ± 1°C, 50 to 60 %RH and a 16:8 L:D photoperiod maintained by LED grow lights (SANlight FLEX 20).

### Method details

#### Pre-experimental treatments

In the experiments, we assayed the behavior of adult *P. persimilis* females that had been exposed to IGP risk during the larval and early protonymphal stage (hereafter called IGP-experienced) or not (hereafter called IGP-naive). All experimental animals had the same genetic background and only differed in early-life treatments. To obtain eggs giving rise to experimental animals, gravid predator females were randomly withdrawn from the rearing and placed in groups of 15 into detached bean leaf arenas harboring mixed spider mite stages. Each detached leaf arena consisted of a primary leaf of common bean, placed abaxial side up on a sheet of moist filter paper on a water-soaked foam block (14 × 14 × 4 cm) inside a plastic box (20 × 20 × 6 cm) half-filled with water. Tissue paper was wrapped around the petiole and the edges of the leaf to maintain turgidity and prevent the predator females from escaping. Eggs laid by the predators within 24 h were collected and transferred in groups of 15 to new detached leaf arenas (day 1), each harboring five adult spider mite females and the eggs produced by them during the 3 preceding days. On the next day (day 2), half of these arenas each received two gravid females of the IG predator *Amblyseius andersoni*, whereas the other half of these arenas were left free of *A. andersoni*. *Phytoseiulus persimilis* larvae started to hatch on day 3 and the first protonymphs appeared on day 4. On day 4, the *A. andersoni* females and any eggs laid by them in the arenas were removed. Thus, one-half of *P. persimilis* (IGP-experienced) grew up in arenas with physical presence of the IG predators for two days, while they were in the egg, larval and early protonymphal stage, but without IG predators for the remainder of their development, which lasted another three to four days; the other half of *P. persimilis* (IGP-naive) grew up, from egg to adult, in arenas without IG predators. This set-up provides for random IGP.^18^^,^^20^ The arenas were checked daily for survival and developmental progress of *P. persimilis*. All *P. persimilis* were left to develop and reach adulthood and mate in their respective arenas, with total development, from egg to adult and mating, lasting about seven to eight days. The spider mites were present in surplus, to avoid food competition, and more than enough for all *P. persimilis* to reach adulthood and to mate in their respective arena. Gravid *P. persimilis* females, discernible by their extended body size, were used in experiments assessing their personalities in boldness and aggressiveness. We used different individuals and ran separate assessments for boldness and aggressiveness to avoid inadvertent within-individual cross-test influences on behavioral trait expressions; for example, exposure to risky sites could make the experimental animals more aggressive in the next test, which might then obscure or change the latent effects of early-life experiences on personality expression after becoming adult.

#### Personality assays

Each IGP-experienced and IGP-naive *P. persimilis* female was tested for consistency in either boldness or aggressiveness. For each behavioral trait, we conducted three tests, each representing a different context. In between personality assays, each predator female was housed individually in a closed acrylic cage and provided with mixed spider mite stages as prey (dubbed home cage). Each home cage consisted of a circular cavity (15 mm Ø × 3 mm high), drilled into an acrylic plate, closed by a fine mesh at the bottom and a removable glass slide, fixed by binder clips, on the upper side.^33^ Predators were transferred between home cages and test cages by using a fine (size 0), moistened marten-hair brush. Boldness was assessed via the residence choice of the predators in T-maze-shaped microcosms that contained safe (void of IG predator cues) and risky (contaminated with IG predator cues) sites. Aggressiveness was assessed via the propensity of the predators to cannibalize (i.e., to kill and eat) larvae. Cannibalism is considered the most extreme form of conspecific aggression.^12^^,^^14^^,^^34^^,^^35^ Additionally, we quantified reproduction of the predators via the number of eggs produced in the test cages. In total, we tested 43 IGP-naive and 31 IGP-experienced females for boldness and 71 IGP-naive and 58 IGP-experienced females for aggressiveness.

To start the boldness assays, IGP-experienced and IGP-naive females were withdrawn from their respective leaf arenas and individually placed in their home cages. On the next day (day B_1_), the third day (day B_3_), and the fifth day (day B_5_), each female was subjected to a boldness test, by assessing the female’s residence preference in an acrylic T-maze cage harboring safe and risky sites. In between boldness assays, the females were returned to their home cages. Each T-maze cage consisted of two large (15 mm Ø) and one small (5 mm Ø) cavity that were connected with each other by a T-shaped aisle (2 mm wide), all drilled into an acrylic plate (80 × 35 × 3 mm) closed at the bottom by a fine mesh and on the upper side by a removable glass slide fixed by binder clips.^36^ The large cavities were located at either end of the horizontal aisle of the T and represented sites with and without IG predator cues; the small cavity was located at the bottom end of the vertical aisle of the T and served as release point of the predator female. In each boldness assay, one large cavity had been inhabited by an IG predator female for 12 h before starting the assay, and was thus contaminated with her traces, such as metabolic waste products and chemical footprints, and was accordingly considered a risky site. The other large cavity was free of IG predator traces and was considered a safe site. To evaluate boldness across contexts,^7^ we used three different IGP scenarios. In the 1^st^ boldness assay, the risky site contained traces of the familiar IG predator *A. andersoni*, in the 2^nd^ boldness assay, the risky site contained traces of the unfamiliar IG predator *N. californicus*, in the 3^rd^ boldness assay, the risky site contained traces of the IG predator *N. californicus* plus traces of spider mites *T. urticae* (each IG predator had been offered about five mobile spider mites inside the cage). All IG predator females and living and dead spider mites including their eggs, feces and silk threads were removed from the risky sites before starting the boldness assays. Residence of the female in the safe and risky site was monitored immediately after release and then every 20 min for 2 h. Number and location of eggs laid by the female during the assay were recorded.

To start the aggressiveness assays, IGP-experienced and IGP-naive females were withdrawn from their respective leaf arenas and individually placed in their home cages. On the next day (day A_1_), each female was subjected to the 1^st^ aggressiveness test, by placing a satiated female in an acrylic test cage that had been previously loaded with a conspecific larva. Conspecific larvae offered as potential prey were unrelated to the experimental female. These larvae emerged from eggs that were randomly collected from another rearing unit and left on blank detached leaves until hatching. The cages were monitored every 30 min for 7 h and then again after 24 h for the occurrence and time of cannibalism of the larva (involving attack, killing and eating) and eggs laid by the female. *P. persimilis* females can discriminate kin and non-kin and refrain from cannibalizing kin including own eggs.^14^^,^^37^ After 24 h, the females were taken out from the test cages and returned to their home cages (day A_2_). After 8 h in their home cages, the females were transferred to new test cages and held without prey until the next day (day A_3_). After 14 h starvation, which was chosen to provide for a different context, each female was offered one conspecific larva as prey (2^nd^ aggressiveness test). Cages were monitored every 30 min for the occurrence and time of cannibalism of the larva and eggs laid by the female for 7 h. Subsequently, the females were returned to their home cages. On the next day (day A_4_), after 14 h in their home cages, the females were singly transferred to new test cages that had been previously inhabited for 24 h by an IG predator female *Neoseiulus californicus* (without any food), and were thus contaminated with their traces such as metabolic waste products and chemical footprints, and were each loaded with one conspecific larva. The IG predator and any eggs laid by them were removed before tests. The cages were monitored every 30 min for 7 h and then again after 24 h for the occurrence and time of cannibalism of the larva and eggs laid by the female (3^rd^ aggressiveness test). Cannibalism occurring within the first observation window of 7 h was categorized as day 1 cannibalism, cannibalism occurring between 7 h and 24 h as day 2 cannibalism.

To enable evaluating within-group personality composition, personality types in boldness were categorized from −3 (extremely shy) over 0 (neutral) to +3 (extremely bold), based on the consistency in residence preference (judged by the proportional time) in the safe or risky site in the three tests (−3 for 3 times > 50% time in safe up to +3 for 3 times > 50% in risky; Table S1). Personality types in aggressiveness were categorized from 0 (not aggressive) to 9 (extremely aggressive), based on the consistency in occurrence and timing of cannibalism in the three tests (0 for 3 times no cannibalism up to 9 for 3 times cannibalism on day 1; Table S1). Marginal values indicate highly consistent types (low-end values for consistently extremely docile or shy, high-end values for consistently extremely bold or aggressive), whereas intermediate values indicate more plastic types.

### Quantification and statistical analysis

All statistical analyses were conducted using IBM SPSS Statistics 28.0.1.0. Survival to adulthood, in total and of females only, in the leaf arenas used for development and IGP conditioning of the experimental animals was compared between treatments (IGP risk or not) by generalized linear models (GLM; binary distribution, counts of events). To verify random IGP, we calculated the Pearson product-moment correlation coefficients between the number of individuals surviving in the conditioning arenas and their personality scores in boldness and aggressiveness. Mean boldness (proportion of time in risky site; normal distribution) and mean aggressiveness (larva cannibalized no/yes_day1/yes_day2; multinomial distribution) in each of the three tests were compared between treatments and among tests by separate generalized estimating equations, using test sequence as inner subject variable to account for inter-test dependency. The total number of eggs (Gamma distribution, loglinear link) produced in the three tests of aggressiveness and of boldness, respectively, were compared between treatments by GLM. Distribution of the eggs in the safe and risky sites of the boldness assays was compared within treatments by Wilcoxon signed rank tests. Personality expression, i.e., behavioral consistency (also called repeatability) over three tests representing different contexts, in boldness (proportion of time in risky site; average measure) and aggressiveness (cannibalism yes/no and attack latency with a ceiling time for non-attacking predators – log-transformed before tests; the ceiling time was twice the maximum attack latency in each test; single measure) was determined by intraclass correlation coefficients (ICC; two-way random, consistency). ICCs were calculated for all predators from both early-life treatments (IGP-naive and IGP-experienced) and separately for each treatment. Repeatability in binary-coded cannibalism propensity (yes/no) in three tests^38^ was additionally analyzed by Fleiss’ Kappa and ‘rptR’ for binary data.^39^^,^^40^ Within- and among-individual variances were checked to pinpoint the causes of treatment-induced changes in ICCs. Within-group personality composition was compared between treatments (IGP-naive and IGP-experienced) by GLM using the personality scores in aggressiveness (Poisson distribution; loglinear as link function) and boldness (multinomial distribution; cumulative probit as link function) as dependent variables. The adaptive values of personality types in boldness and aggressiveness were examined by linear regression of the pooled number of eggs laid during the test procedures (as a proxy of short-term fitness) on the personality scores along the shy-bold axis and aggressiveness continuum, respectively. Use of linear regression was based on the best fitting model determined by the curve estimation procedure. Boxplots in Figures 2 and 4 were created by BoxPlotR,^41^ Figures 3, 5, and 6 were created by IBM SPSS Statistics 28.0.1.0.
