# Supplementary material for: Early-life intraguild predation risk produces adaptive personalities in predatory mites
Source: iScience. 2024 Feb 1;27(3):109065. doi: 10.1016/j.isci.2024.109065 (PMC10864791; doi:10.1016/j.isci.2024.109065)
Supplement: Document S1. Table S1 [file mmc1.pdf]

iScience, Volume *27*

Supplemental information

Early-life intraguild predation risk produces adaptive personalities in predatory mites

Peter Schausberger, Thi Hanh Nguyen, and Mustafa Altintas

Table S1. Categorization of personalities in boldness and aggressiveness; related to figures 5 and 6.

Personality score in boldness	Percent time in risky site in 3 successive tests[a]
-3	<50,<50,<50
-2	<50,<50,=50
-1	<50,<50,>50/<50,=50,=50
0	<50,>50,=50/=50,=50,=50
1	>50,>50,<50/>50,=50,=50
2	>50,>50,=50
3	>50,>50,>50
**Personality score in aggressiveness**	**Cannibalism in 3 successive tests[a,b]**
0	0,0,0
1	0,0,2
2	0,0,1
3	2,0,2
4	0,1,2
5	0,1,1
6	2,2,2
7	2,1,2
8	1,1,2
9	1,1,1

[a]Sequence of values is interchangeable

[b]0 for no cannibalism; 1 for cannibalism on day 1; 2 for cannibalism on day 2
